# Role of Sphingosine Kinase 1 in Glucolipotoxicity-Induced Early Activation of Autophagy in INS-1 Pancreatic β Cells

**DOI:** 10.3390/cells13070636

**Published:** 2024-04-05

**Authors:** Nicolas Coant, Karima Rendja, Lara Bellini, Mélissa Flamment, Jeannine Lherminier, Bernard Portha, Patrice Codogno, Hervé Le Stunff

**Affiliations:** 1 Unité BFA, Université Paris Cité, CNRS UMR 8251, 75006 Paris, France; nicolas.coant@stonybrookmedicine.edu (N.C.); bernard.portha@u-paris.fr (B.P.); 2 Department of Pathology and Stony Brook Cancer Center, Stony Brook University Renaissance School of Medicine, Stony Brook, NY 11794, USA; 3 Inserm, UMR-S 872, Centre de Recherche des Cordeliers, 75006 Paris, France; 4 INRA, UMR1347 Agroécologie, ERL CNRS 6300, Plateforme DImaCell, Centre de Microscopie INRA/Université de Bourgogne, 21065 Dijon, France; 5 INSERM U1151-CNRS UMR 8253, Institut Necker Enfants-Malades, University Paris Descartes, 75006 Paris, France; 6 CNRS UMR 9197, Institut des Neurosciences Paris-Saclay, Saclay, University Paris, 91400 Saclay, France

**Keywords:** autophagy, sphingosine kinase 1, sphingosine-1-phosphate, ceramides, type 2 diabetes, gluco-lipotoxicity, cell death, pancreatic β cells

## Abstract

Insulin-producing pancreatic β cells play a crucial role in the regulation of glucose homeostasis, and their failure is a key event for diabetes development. Prolonged exposure to palmitate in the presence of elevated glucose levels, termed gluco-lipotoxicity, is known to induce β cell apoptosis. Autophagy has been proposed to be regulated by gluco-lipotoxicity in order to favor β cell survival. However, the role of palmitate metabolism in gluco-lipotoxcity-induced autophagy is presently unknown. We therefore treated INS-1 cells for 6 and 24 h with palmitate in the presence of low and high glucose concentrations and then monitored autophagy. Gluco-lipotoxicity induces accumulation of LC3-II levels in INS-1 at 6 h which returns to basal levels at 24 h. Using the RFP-GFP-LC3 probe, gluco-lipotoxicity increased both autophagosomes and autolysosmes structures, reflecting early stimulation of an autophagy flux. Triacsin C, a potent inhibitor of the long fatty acid acetyl-coA synthase, completely prevents LC3-II formation and recruitment to autophagosomes, suggesting that autophagic response requires palmitate metabolism. In contrast, etomoxir and bromo-palmitate, inhibitors of fatty acid mitochondrial β-oxidation, are unable to prevent gluco-lipotoxicity-induced LC3-II accumulation and recruitment to autophagosomes. Moreover, bromo-palmitate and etomoxir potentiate palmitate autophagic response. Even if gluco-lipotoxicity raised ceramide levels in INS-1 cells, ceramide synthase 4 overexpression does not potentiate LC3-II accumulation. Gluco-lipotoxicity also still stimulates an autophagic flux in the presence of an ER stress repressor. Finally, selective inhibition of sphingosine kinase 1 (SphK1) activity precludes gluco-lipotoxicity to induce LC3-II accumulation. Moreover, SphK1 overexpression potentiates autophagic flux induced by gluco-lipotxicity. Altogether, our results indicate that early activation of autophagy by gluco-lipotoxicity is mediated by SphK1, which plays a protective role in β cells.

## 1. Introduction

Type 2 Diabetes (T2D) is a chronic metabolic disease characterized by hyperglycemia and insulin resistance [1]. Long-standing T2D eventually results in the decrease in secretion of insulin by pancreatic β cells and the inability of peripheral tissues to store glucose in response to insulin [2]. The presence of chronic hyperglycemia induces many deleterious effects on the pancreas itself, with reduction in the β cell mass being chief among them. This phenomenon is called glucotoxicity [3]. It has been observed in many type 2 diabetic patients that hyperglycemia is associated with high lipid concentrations in the plasma. It is now currently understood that hyperlipidemia in combination with hyperglycemia induces β cell dysfunction and apoptosis [4,5]. It has also been described that high glucose and high lipid exposure inhibit insulin gene transcription in rat and human islets [6,7,8]. Importantly, the chronic negative effects of free fatty acids (FFAs) on β cell function and viability are potentiated by the presence of hyperglycemia, a phenomenon that has been termed “gluco-lipotoxicity” [9].

Macroautophagy (hereafter referred to as autophagy) has first been described in cells as means to recycle damaged organelles in order to maintain cell homeostasis [10]. Indeed, an important role of autophagy is to transform nutrients from endogenous sources for use by starved cells. In mammals, autophagy is a regulated process that is activated in cell growth, development and homeostasis [11], for which it can play a protective role against various types of injuries and as an anti-aging mechanism [12], while also contributing to cellular defense against pathogens [13]. Autophagy is a sequential process which starts with the formation of a lipidic structure called phagophore close to the endoplasmic reticulum (ER) [14]. This structure then sequesters cytoplasmic material in a selective or nonselective manner to form autophagosomes [15]. Among the large numbers of proteins involved in this phenomenon, LC3, a cytoplasmic protein homologue of the yeast protein, ATG8, undergoes a processing and a covalent conjugation to the phospholipid, phosphatidylethanolamine. This modification leads sequentially to LC3-I and LC3-II [16]. The last step of autophagy is the fusion of the autophagosome to the lysosome leading to a structure called an autolysosome that results in the degradation of the sequestrated cytoplasmic material by lysosomal proteases [17].

It has been recently proposed that autophagy plays a central role for normal β cell function and survival and that a dysregulation could contribute to β cell failure in T2D [18,19,20]. Indeed, Choi et al. showed that FFAs appear to stimulate the conversion of LC3-I to LC3-II, as well as the set up of autophagosomes and autolysosomes in β cells [18]. In these conditions, autophagy protects against palmitate-induced β cell death [21]. Importantly, using a mouse model of β cell-specific loss of autophagy, Ebato et al. showed that the induction of β cell autophagy by a high fat diet is a crucial element for protecting β cells from lipotoxicity [19]. However, the induction of autophagy by FFAs in β cells has been recently questioned by studies showing that FFAs induced LC3-II accumulation through a suppression of autophagic turn-over in β cells [22,23]. It appears from these studies that, in vitro, long-term exposure to fatty acids and glucose inhibited autophagic flux in β cells. In contrast, short-term exposure to palmitate appeared to stimulate autophagic flux [18]. Recently, Biden and colleagues elegantly showed that an 8–10 weeks high fat diet increased in vivo and ex vivo autophagic flux in β cells [24]. Therefore, it is conceivable that the regulation of autophagy by FFAs is as an adaptive response of β cells for survival under stressful conditions.

At present, few studies have explored the mechanisms involved in palmitate-stimulated autophagy functions in β cells. A recent study showed that activation of the JNK pathway could play a role in the stimulation of β autophagy induced by palmitate [21]. The molecular process underlying the pathogenesis of gluco-lipotoxicity in pancreatic β cells seems to involve sphingolipid metabolism [4,25]. We and others have shown that ceramides are crucial mediators of FFA-induced β cell dysfunction and apoptosis [4,25]. More recently, activation of palmitate-induced sphingoid base-1-phosphate formation in β cells has been shown to play a protective role against palmitate-induced apoptotic β cell death [5]. Interestingly, ceramide and sphingoid base-1-phosphates both have been shown to induce autophagy [26,27]. Moreover, inhibition of sphingosine kinase 1 (SphK1), the enzyme responsible for sphingoid base-1-phosphates [28] by siRNA, prevented autophagy and promoted cell death induced by starvation. These results suggest that autophagy is a novel mechanism of S1P’s prosurvival response [26]. The precise role of induction of autophagy by ceramide is still unclear. For example, despite the fact that ceramide is a known pro-apoptotic agent, ceramide-dependent autophagy still appears to have a cytoprotective effect against the induction of apoptosis of primary rat hepatocytes, apoptosis induced by bile + MEK inhibitors, and cancer cells by sorafenib and vorinostat [29].

In the current study, we examine the role of palmitate metabolism in gluco-lipotoxicity-induced β cell autophagy. Using different approaches, we found that palmitate induced an increase in autophagic turn-over in β cells. Our data also revealed that palmitate metabolism is required but that de novo ceramide synthesis is not involved, whereas sphingoid base-1-phosphate synthesis contributes to β cell autophagy induced by gluco-lipotoxicity.

## 2. Materials and Methods

### 2.1. Materials

Tissue culture medium was obtained from Lonza. Palmitate, fatty-acid-free BSA, bafilomycin A1 and C6-ceramide were obtained from Sigma–Aldrich. FB1, sphingosine kinase inhibitor (SKI), fumonisin B1 (FB1), D,L-threo-1-phenyl-2-palmitoylamino-3-morpholinopropan-1-ol (PPMP), and sphingosine-1-phosphate were from Biomol. Apo-ONE^®^ Homogenous Caspase-3/7 Assay kits were from Promega. All solvents were from Merck Eurolab or Fisher Scientific. Ceramides and C17-sphingosine were from Avanti Polar Lipids. Anti-LC3 (Cell Signaling, clone 2775, dilution 1/1000 for Western-blot; clone D11, dilution 1/500 for immunofluorescence), and anti-β-actin (Sigma-Aldrich, St. Louis, MO, USA, clone 8H10D10, dilution 1/5000) antibodies were from Sigma-Aldrich. Secondary alexa fluor antibodies and ProLong™ Gold Antifade Mountant with DAPI were from Invitrogen™, Waltham, MA, USA.

### 2.2. Cell Culture Conditions

Rat insulinoma INS-1 β cells (clone 368), kindly provided by Merck–Serono, were grown in RPMI 1640 medium buffered with 10 mM Hepes containing 10% (*v*/*v*) FBS (fetal bovine serum), 2 mM L-glutamine, 1 mM sodium pyruvate, 50 μM 2-mercaptoethanol, and 100 units/mL penicillin/streptomycin. FFAs were added to the cells as a conjugate with fatty-acid-free BSA as described previously [4]. Cells were stably transfected with a vector, V5-SphK1, or HA-CerS4 and cultured in medium containing 1 g of G418/liter (for SphK1) or 0.1 mg/mL of hygromycin (for CerS4) as previously described [4,28]. INS-1 β cells were transiently transfected with RFP-GFP-tagged LC3 plasmid construction [30] using Lipofectamine™ LTX (Thermo Fisher Scientific, Waltham, MA, USA). Transfection efficiencies were between 40 and 70% for INS-1 β cells.

### 2.3. Electron Microscopy

Cells were fixed with 2.5% glutaraldehyde buffered in 0.1 M sodium cacodylate, pH 7.4 at room temperature for 1 h. After washing, the cells were post-fixed in 1% OsO4 solution for 1 h at room temperature, rinsed and dehydrated in an ethanol gradient (70% to 100%, 10 min for each bath). Absolute ethanol was replaced by propylene oxide. The samples were infiltrated by epoxy resin (R1165, Agar scientific, Stansted, UK) mixed to propylene oxide (50–50%) overnight, which was then followed by three baths with pure epoxy resin (during 1 day). Cells were polymerized at 60 °C during 18 h. Ultra-thin sections (70 nm) cut with an ultra-microtome (Leica™ UC6, Nanterre, France) were stained with uranyl acetate (20 min) and Reynolds lead citrate (2 min). Sections were observed at 80 kV, in a TEM Phillips™ (Waltham, MA, USA) Tecnai 12 equipped with an Olympus™ Keenview CCD camera.

### 2.4. Immunofluorescence Analysis

Cells were fixed with 4% PFA solution for 20 min and washed with PBS. After saturation in PBS containing 5% goat serum and 0.01% saponin, cells were incubated with a polyclonal anti-LC3 antibody and with a monoclonal anti-V5 antibody when relevant. Cells were subsequently incubated with the appropriate secondary antibody. Coverslips were mounted on glass slides using an anti-fade kit (VectaShield mounting medium with DAPI) and examined by confocal microscopy. For RFP-GFP-LC3 images analysis, cells were fixed with 4% PFA and washed with PBS. Images were collected by a Ziess™ (Munich, Germany) Axiovison laser scanning microscope and a 63× lens. Quantitative image analysis was performed using the Zen2009 image processing software. To avoid fluorescence crossover between the channels, Alexa Fluor488 and Texas red images were collected separately using the appropriate laser excitation (488 nm and 568 nm, respectively) and then merged. Dots were manually counted in a double-blind manner from a minimum of 10 fields per condition. The number of LC3 dots, RFP-GFP-LC3 dots (yellow), and RFP-LC3-dots (red) were determined by directly counting over 100 cells for each experimental condition.

### 2.5. Measurement of Caspase-3/7 Activity

Caspase-3/7 activity assays were performed as previously described using the Promega Apo-ONE Homogeneous Caspase-3/7 Assay kit. Briefly, lysis buffer with the fluorogenic Z-DEVD-R110 substrate was added and fluorescence was measured over a 120 min period using a Fluostar plate reader set at 37 °C (with excitation at 485 nm and emission at 530 nm). Caspase-3/7-specific activity was shown as the slope of the kinetic in arbitrary units. Each experimental condition tested was performed in triplicate.

### 2.6. Western Blotting

Equal amounts of proteins were separated by SDS/PAGE (10% or 15% gels) and then transblotted on to PVDF. Blots were probed with either a polyclonal anti-LCII antibody or a monoclonal anti-β-actin antibody. Immunoreactive bands were visualized by enhanced chemiluminescence with appropriate horseradish peroxidase-conjugated secondary antibody (Jackson ImmunoResearch Laboratories™, West Grove, PA, USA) and the ECL Plus Western Blotting Detection System (Amersham™, Saint-Germain en Laye, France). Image acquisition was performed with the LASS 4000 detection system (Fuji™, Chicago, IL, USA).

### 2.7. Analysis of Ceramides Levels

Ceramide levels in cell extracts were measured by the diacylglycerol (DAG) kinase enzymatic method as previously described [29]. Briefly, aliquots of cellular lipid extracts were resuspended in 7.5% (*w*/*v*) octyl-β-D-glucopyranoside/5 mM cardiolipin in 1 mM DETPAC/10 mM imidazole (pH 6.6). The enzymatic reaction was initiated by the addition of 20 mM DTT, 0.88 U/mL *E. coli* DAG kinase, 5 μCi/10 mM [γ-32P]ATP, and the reaction buffer (100 mM imidazole (pH 6.6), 100 mM NaCl, 25 mM MgCl2, and 2 mM EGTA). After 1 h at room temperature, lipids were extracted with chloroform/methanol/HCl (100:100:1, *v*/*v*) and 1 M KCl. [γ-32P]-ceramide phosphate was separated by TLC with chloroform/acetone/methanol/acetic acid/water (10:4:3:2:1, *v*/*v*) and quantified with a phosphorimager (Amersham, Saint-Germain en Laye, France). Known amounts of bovine ceramide standards were included in each assay. Ceramide levels are expressed as fmol by nmol of phospholipid (PL) levels. Each measurement was conducted in duplicate.

### 2.8. PCR Analysis of XBP1 Splicing

Total RNA was isolated from INS-1 β cells using the RNAeasy mini kit (Qiagen, Courtaboeuf, France). Total RNA (4 µg) from each sample was reverse transcribed with 40 U of M-MLV Reverse Transcriptase (Invitrogen, Waltham, MA, USA) using poly T primers. To quantify relative expression levels of U-XBP1/S-XBP1s, RT-PCR analysis was performed using PCR SuperMix (Invitrogen). Human XBP1 primer sequences were as follows: 5′-CCTGGTTGCTGAAGAGGAGG-3′ and 5′-CCA TGGGGAGATGTTCTGGAG-3′. The PCR conditions were as follows: 95 °C for 10 min, followed by 40 cycles at 95 °C for 10 s, 60 °C for 10 s, and 72 °C for 10 s. PCR products were analyzed on a 3.5% agarose gel.

### 2.9. Statistical Analysis

Data were expressed as means ± S.E.M. Significance was assessed by the Student’s *t* test. *p* values less than 0.01 were considered as significant.

## 3. Results

### 3.1. Palmitate with High Glucose Stimulate LC3-II Accumulation in INS-1 β Cells

**Figure 1:**
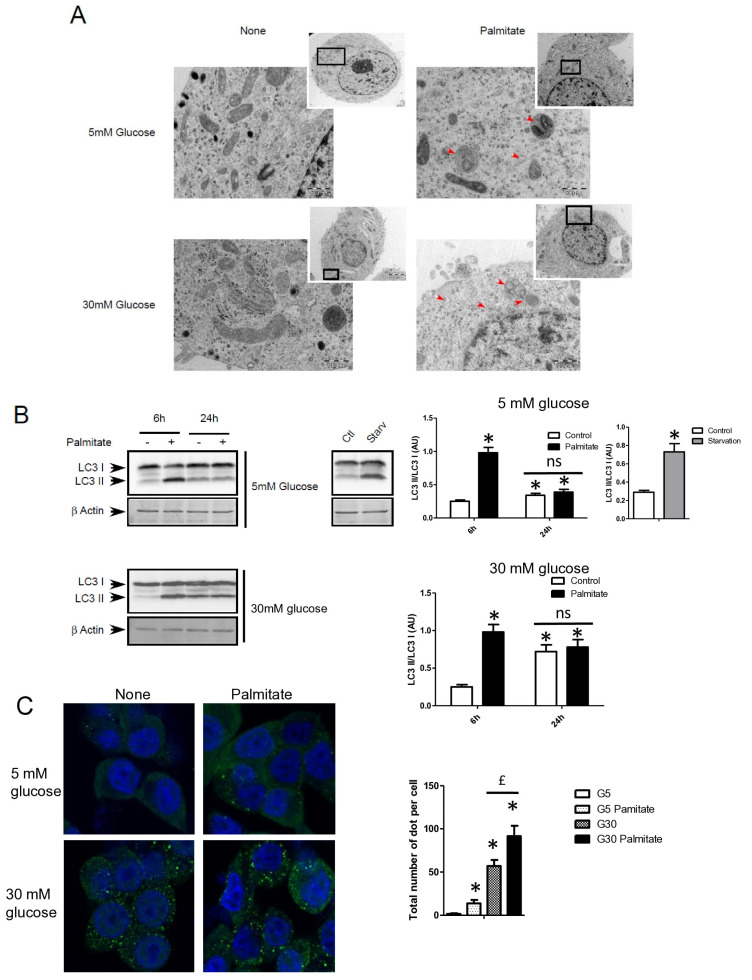

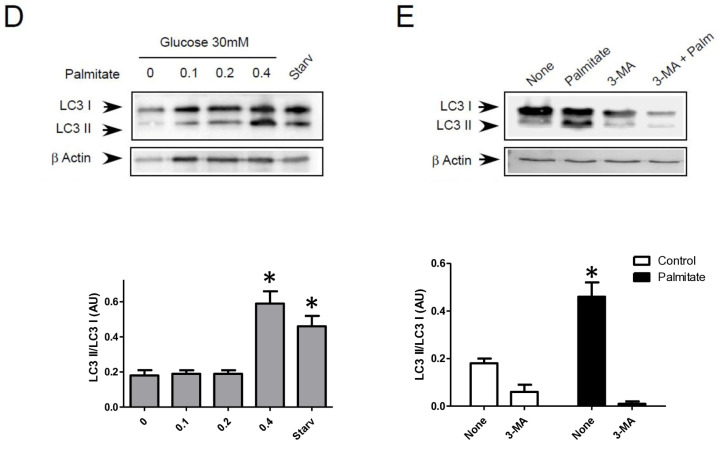
Palmitate with high glucose concentrations induces autophagy in INS-1 β cells. (**A**) Representative transmission electronic microscopy image analysis of INS-1 β cells exposed to low and high concentrations of glucose (5 mM or 30 mM) with or without 0.4 mM palmitate during a 24 h period. Autophagosomes are intracellular formations characterized by a double membrane that contains cytoplasmic organelles (arrow head). (**B**) Representative Western blot analysis of LC3-I and LC3-II levels in INS-1 β cells after exposure to 0.4 mM of palmitate for 6 h and 24 h with low glucose concentration (5 mM, upper panel) and high concentration glucose (30 mM, lower panel). Control and starvation conditions used as negative and positive control on LC3-II levels. Scanning densitometry was performed to quantify changes in LC3-II abundance in cell lysates normalized with LC3-I levels. Results represented as LC3-II/LC3-I (AU) are mean +/− SEM (*n* = 3). * Significant change *p* ≤ 0.01 relative to the control; ns: non-significant. (**C**) Representative confocal microscopy image analysis of INS-1 β cells exposed to 5 mM or 30 mM glucose with or without 0.4 mM palmitate for 24 h. Cells were immune stained with anti-LC3 antibody (green), and the nucleus was counter stained with DAPI (blue). For each condition, the total number of green dots (LC3-II) were counted per cell and reported. The mean of the number of red dots counted per cell in each condition was calculated. Results are the mean +/− SEM (*n* = 3). * Significant change *p* ≤ 0.01 relative to the control (glucose 5 mM glucose), ^£^ significant change *p* ≤ 0.01 relative to the glucose 30 mM. (**D**) Representative Western blot analysis of LC3-I and LC3-II levels in INS-1 β cells after exposure to increased concentrations of palmitate (0.1, 0.2, 0.4 mM) with 30 mM of glucose for 6 h. Scanning densitometry was performed to quantify changes in LC3-II abundance in cell lysates normalized with LC3-I levels. Results represented as LC3-II/LC3-I (AU) are mean +/− SEM (*n* = 3). * Significant change *p* ≤ 0.01 relative to the control (absence of palmitate). (**E**) Representative Western blot analysis of LC3-I and LC3-II levels in INS-1 β cells after 6 h exposure to 0.4 mM of palmitate with or without autophagic inhibitor 3-MA (10 μM). Scanning densitometry was performed to quantify changes in LC3-II abundance in cell lysates normalized with LC3-I levels. Results represented as LC3-II/LC3-I (AU) are the mean +/− SEM (*n* = 3). * Significant change *p* ≤ 0.01 relative to the control (absence of palmitate).

We first examined the presence of autophagic structure in electron microscopy (EM) images obtained from INS-1 β cells incubated with palmitate in the presence of various concentrations of glucose for 12 h (Figure 1A). The ultrastructure observed by EM of INS-1 β cells treated with 5 or 30 mM of glucose did not show the presence of vacuolated structures representative of autophagy (Figure 1A). In contrast, the addition of 0.4 mM of palmitate in these latter conditions induced the appearance of vacuoles engulfing cytoplasmic organelles in INS-1 β cells, a characteristic of autophagy (Figure 1A, arrows). We observed the presence of several characteristic findings of vacuole structures, such as vacuoles with double membranes and dark debris, and vacuoles with organelles and multi-lamellar structures (Figure 1A, arrows). In order to prove the activation of an autophagic process by palmitate, we also evaluated the presence of the conversion of LC3-I to LC3-II in INS-1 β cells. As a positive control for autophagy, we found that starvation of INS-1 β cells for 2 h resulted in the conversion of LC3-I to LC3-II (Figure 1B, right panel; Supplementary Figure S1A). We also found that palmitate induced conversion of LC3-I to LC3-II at concentrations of both 5 and 30 mM of glucose after 6 h of treatment, but disappeared at 24 h (Figure 1B left panel; Supplementary Figure S1A). Moreover, in agreement with a previous study [31], high glucose levels started to increase the LC3-II levels at 24 h in INS-1 β cells. High glucose levels were not able to increase the ratio of LC3-II/LC3-I induced by palmitate at 6 h (Figure 1B quantifications). However, palmitate increased the ratio of LC3-II/β-actin at 6 h (Supplementary Figure S1A). Using confocal fluorescence microscopy, we found that palmitate at low glucose levels induced the re-localization of cytoplasmic LC3-I into a punctuated pattern at low glucose levels (Figure 1C), reflecting the presence of LC3-II to the autophagosome membrane [32]. We found that 30 mM of glucose elicited a staining of punctuated LC3-II and slightly potentiated the effect of palmitate by increasing the amount and size of the LC3-II autophagosome membrane (Figure 1C). Palmitate with 30 mM glucose started to increase LC3-II levels at the concentration of 0.4 mM (Figure 1D and Supplementary Figure S1B). In agreement with another previous study [18], we found that inhibition of class III PI3K, a key enzyme involved in autophagosome formation, by 3-methyl-adenine (3-MA) completely inhibited the conversion of LC3-I to LC3-II which was induced by palmitate (Figure 1E and Supplementary Figure S1C). Taking together, these results support the idea that a treatment with palmitate, with high glucose levels, stimulates LC3-II accumulation in INS-1 β cells.

### 3.2. Palmitate with High Glucose Levels Stimulates an Early Autophagy Flux in INS-1 β Cells

**Figure 2:**
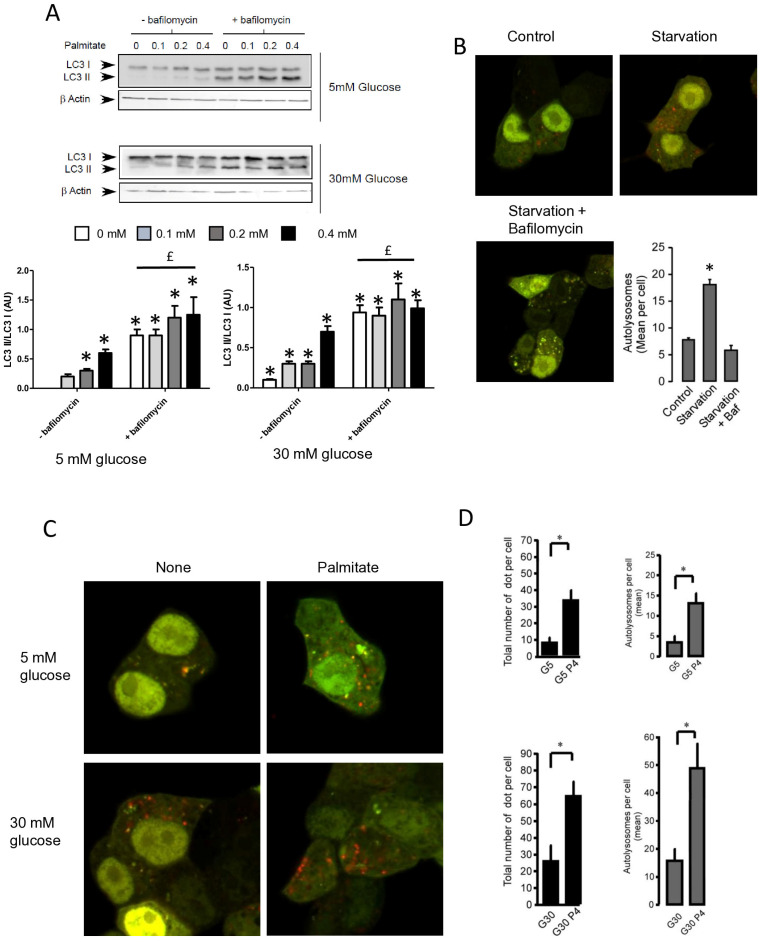
Palmitate with high glucose concentrations induces autophagic flux in INS-1 β cells. (**A**) Representative Western blot analysis of LC3-I and LC3-II expression INS-1 β cells after 6 h exposure to increase concentration of palmitate (0.1, 0.2, 0.4 mM) with or without bafilomycinA1 (200 µM) in presence of low concentration of glucose (5 mM upper panel) or high concentration (30 mM, lower panel). Scanning densitometry was performed to quantify changes in LC3-II abundance in cell lysates normalized with LC3-I levels. Results represented as LC3-II/LC3-I (AU) are mean +/− SEM (*n* = 3). * Significant change *p* ≤ 0.01 relative to the control (absence of palmitate). ^£^ significant change *p* ≤ 0.01 relative to the presence of bafilomycin alone. (**B**) Confocal microscopy analysis of INS-1 β cells transfected with RFP-GFP-LC3, constructed, and then starved for 2 h. Total number of red dots (autolysosomes were counted per cell and reported. The mean of the number of red dots counted per cell in each condition was calculated. Results are the mean +/− SEM (*n* = 3). * Significant change *p* ≤ 0.01 relative to the control. (**C**) Confocal microscopy analysis of INS-1 β cells treated with 5 mM or 30 mM concentration of glucose with or without 0.4 mM palmitate for 6 h. (**D**) For each condition presented in (**C**), total number of dots (yellow and red) and autolysosomes (red dots) were counted per cell and then reported (G5: 5 mM of glucose, G5P4 5 mM of glucose and 0.4 mM of palmitate, G30: 30 mM of glucose, G30P4 30 mM of glucose, and 0.4 mM of palmitate) for 6 h. The mean of the number of red dots counted per cell in each condition was calculated. Results are the mean +/− SEM (*n* = 4). * Significant change *p* ≤ 0.01 relative to the control (G5 or G30).

Autophagosomes are intermediate structures that fuse with lysosomes forming an autolysosome which ultimately results in the elimination of the enclosed materials [32,33]. In this context, the number of autophagosomes observed at a given time will demonstrate the dynamic balance between the autophagosome formation, its conversion into autolysosome, and its final degradation. Two recent studies have suggested that LC3-II accumulation induced by long-term exposure to long-term treatment with palmitate could result from the suppression of autophagic turn-over in β cells [22,23]. Our data showed that LC3-II accumulation, which is induced by short-term exposure to palmitate, is transient in INS-1 β cells (Figure 1A). This suggests, as others have previously proposed [11,18], that this treatment increases the rate of autophagosome formation rather than inhibiting autophagosome fusion. In order to better understand the effect of palmitate on autophagic flux in β cells, we tested the effect of BafilomycinA1 (BafA1) on autophagy induced by palmitate with various concentrations of glucose. BafA1 is a potent inhibitor of the acidification of the lysosome which blocks the fusion between the autophagosome and the lysosome [32]. As a result, the difference in LC3-II amount in absence and presence of BafA1 will represent the LC3 quantity that should be delivered to lysosomes for elimination. As shown in Figure 2A, BafA1 treatment resulted in the strong accumulation of LC3-II (line 1 and 5) at both low and high glucose levels. However, palmitate still increased LC3-II accumulation at low and high glucose levels (Figure 2A and Supplementary Figure S2A). Altogether, these data indicate that autophagic flux is increased during palmitate treatment in INS-1 β cells.

To confirm this result, we visualized autophagic flux with a mRFP-GFP-LC3 tandem construct, for which autophagosomes and autolysosomes will be labeled with yellow (i.e., mRFP and GFP) and red (i.e., mRFP only) signals, respectively, due to the quenching of GFP fluorescence at low pH found in lysosomes [30,34]. We first tested the effect of starvation on mRFP-GFP-LC3 localization in INS-1 β cells (Figure 2B). In agreement, we found that starvation of INS-1 β cells overexpressing mRFP-GFP-LC3 protein for 2 h drastically increased both yellow-labeled autophagosomes and red-labeled autolysosomes (Figure 2B and Supplementary Figure S2B). The addition of BafA1 during the starvation increased the total number fluorescent-labeled vacuoles, but this significantly blunted the accumulation of red-labeled autolysosomes (Figure 2B and supplemental Supplementary Figure S2B), reflecting an inhibition of autolysosome formation. Treatment with 5 mM glucose alone induces few yellow and red dots (Figure 2C), whereas the addition of palmitate for 6 h elicited a large increase in the total number of red and yellow dots (3-fold increase, Figure 2D). This increase was due to an almost 50% increase in red dots (Figure 2D), which supports an increase in the autophagic flux during palmitate treatment. We performed the same experiment with 30 mM of glucose (Figure 2C), which led to (i) an increase in the total number of red and yellow dots (2-fold increase, Figure 2D) and (ii) potentiated accumulation of red dots induced by palmitate (Figure 2C,D). Taken together, these results indicate that 6 h treatment with palmitate and high glucose stimulates autophagic flux in β cells.

### 3.3. Palmitate Induces Autophagy through Its Metabolism but Independently of Its Mitochondrial β-Oxidation in INS-1 β Cells

**Figure 3:**
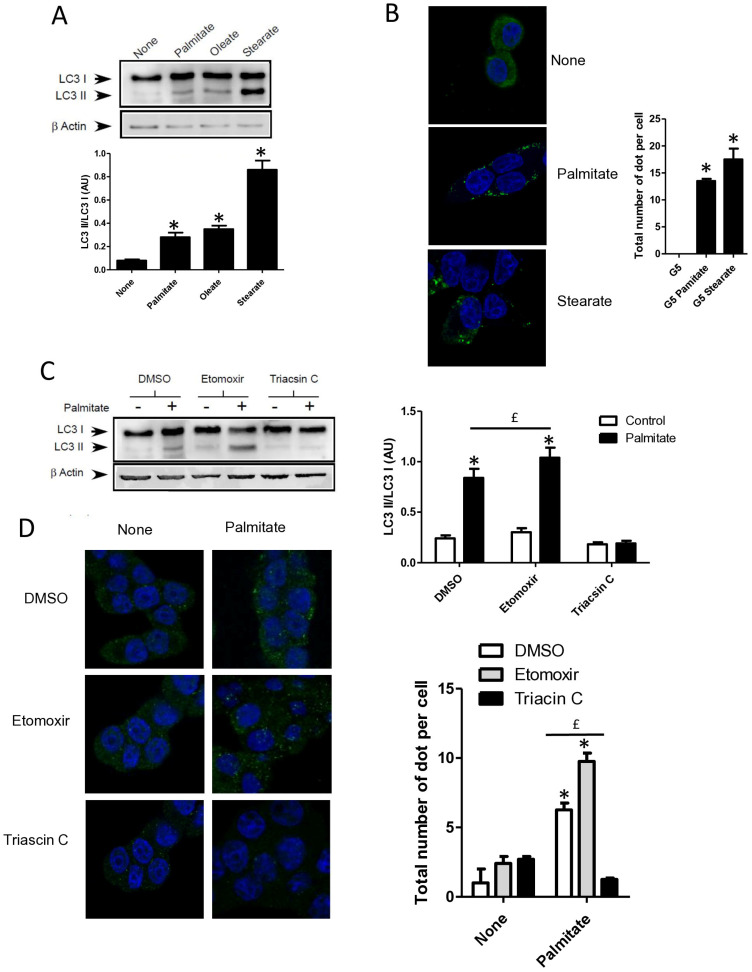

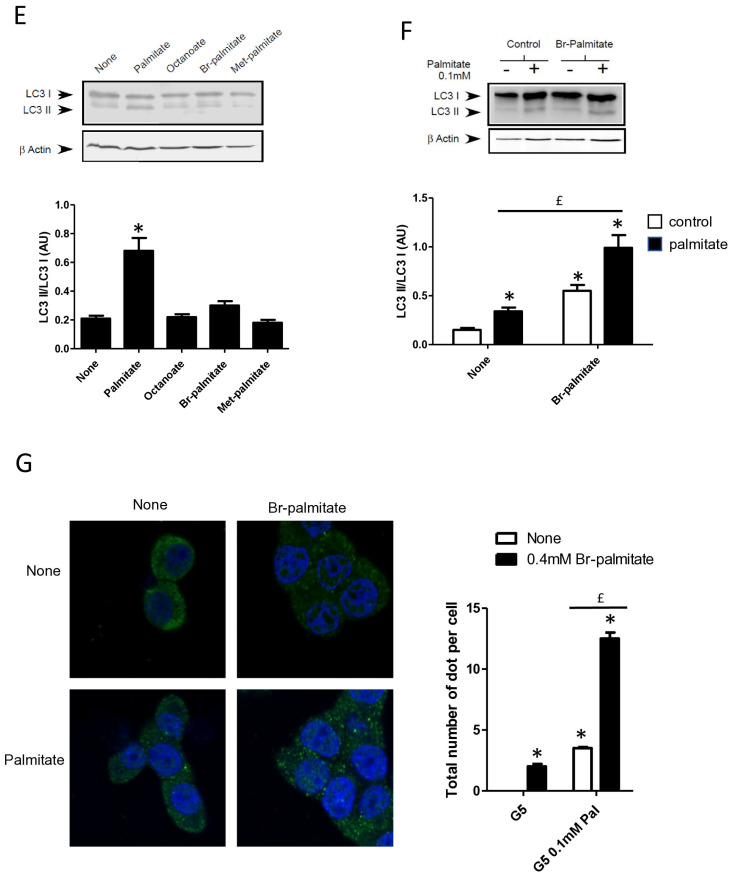
Autophagy induced by palmitate requires its metabolism but is independent of its entry into the β-oxidation pathway in INS-1 β cells. (**A**) Representative Western blot analysis of LC3-I and LC3-II levels in INS-1 β cells treated with 0.4 mM of palmitate, oleate, or stearate for 6 h. Scanning densitometry was performed to quantify changes in LC3-II abundance in cell lysates normalized with LC3-I levels. Results represented as LC3-II/LC3-I (AU) are the mean +/− SEM (*n* = 3). * Significant change *p* ≤ 0.01 relative to the control (absence of fatty acid). (**B**) Confocal microscopy images of INS-1 β cells exposed to 5 mM glucose with or without 0.4 mM palmitate or stearate for 6 h. Cells were immune stained with anti-LC3 antibody (green) and the nucleus was counter stained with DAPI (blue). The mean of the number of green dots counted per cell in each condition was calculated. Results are the mean +/− SEM (*n* = 3). * Significant change *p* ≤ 0.01 relative to the control. (**C**) Representative Western blot analysis of LC3I and LC3II expression INS-1 β cells after exposure to etomoxir or triacsin C at 10 μM in presence or absence of 0.4 mM palmitate with 5 mM glucose for 6 h. Scanning densitometry was performed to quantify changes in LC3-II abundance in cell lysates normalized with LC3-I levels. Results represented as LC3-II/LC3-I (AU) are the mean +/− SEM (*n* = 3). * Significant change *p* ≤ 0.01 relative to the control (absence of palmitate). ^£^ significant change *p* ≤ 0.01 relative to the absence of etomoxir. (**D**) Confocal microscopy images of INS-1 β cells exposed to 5 mM glucose with or without 0.4 mM palmitate for 6 h after exposure to etomoxir (10 μM) or triacsin C (10 μM). Cells were immune stained with anti-LC3 antibody (green) and the nucleus was counter stained with DAPI (blue). The mean of the number of green dots counted per cell in each condition was calculated. Results are the mean +/− SEM (*n* = 3). * Significant change *p* ≤ 0.01 relative to the control. ^£^ significant change *p* ≤ 0.01 relative to the absence of etomoxir. (**E**) Representative Western blot analysis of LC-3I and LC3-II levels in INS-1 β cells treated with palmitate, octanoate, bromopalmitate, or methylpalmitate at 0.4 mM, under condition of 5 mM of glucose for 6 h. Scanning densitometry was performed to quantify changes in LC3-II abundance in cell lysates normalized with LC3-I levels. Results represented as LC3-II/LC3-I (AU) are the mean +/− SEM (*n* = 3). * Significant change *p* ≤ 0.01 relative to the control (absence of palmitate). (**F**) Representative Western blot analysis of LC3-I and LC3-II expression in INS-1 β cells treated with 0.1 mM of palmitate with or without treatment by bromopalmitate (Br-palmitate) at 0.4 mM for 6 h. Scanning densitometry was performed to quantify changes in LC3-II abundance in cell lysates normalized with LC3-I levels. Results represented as LC3-II/LC3-I (AU) are the mean +/− SEM (*n* = 3). * Significant change *p* ≤ 0.01 relative to the control (absence of palmitate). ^£^ significant change *p* ≤ 0.01 relative to the absence of bromopalmitate. (**G**) Confocal microscopy image of INS-1 β cells exposed to palmitate and bromo-palmitate similarly to (**F**). Cells were immune stained with anti-LC3 antibody (green) and nucleus were counter stained with DAPI (blue). The mean of the number of green dots counted per cell in each condition was calculated. Results are the mean +/− SEM (*n* = 3). * Significant change *p* ≤ 0.01 relative to the control. ^£^ significant change *p* ≤ 0.01 relative to the absence of bromopalmitate.

Saturation of fatty acids has been shown to be crucial in the induction of β cell apoptosis [25]. In contrast, unsaturated fatty acids such as oleate have almost no effect on β cell apoptosis [35]. We confirmed that oleate-like palmitate stimulated the conversion of LC3-I to LC3-II (Figure 3A and Supplementary Figure S3A) [22]. Stearate also induced the accumulation of LC3-II (Figure 3A and Supplementary Figure S3A) and induced the re-localization of cytoplasmic LC3-I into a punctuated pattern in INS-1 cells (Figure 3B). Following its entry into cells, palmitate is activated by an acyl-CoA synthase which is either used by mitochondrial β-oxidation to produce energy or transformed into complex lipids such as diacylglycerol or ceramides. Palmitate could also act as a potent agonist of the G protein coupled receptor GPR40/FFAR1 which has a controversial role in β cell lipotoxicity [36,37]. In order to determine the role of palmitate metabolism in induction of autophagy in β cells, we first treated cells with triascin C, an inhibitor of acyl-coA synthase or etomoxir, an inhibitor of mitochondrial β-oxidation. We show that triascin C completely blunted the conversion of LC3-I to LC3-II (Figure 3C and Supplementary Figure S3B) or the formation of autophagosome (Figure 3D) induced by palmitate supporting the idea that fatty acids should be transported into INS-1 cells and be activated in order to stimulate autophagy. In contrast, treatment with etomoxir potentiated the activation of autophagy by palmitate, visualized either with LC3-II accumulation and LC3 puncta (Figure 3C,D, Supplementary Figure S3B). We also found that neither octanoate, which is readily oxidized, nor methyl-palmitate, which enters in the cell but is not metabolized, induces the conversion of LC3-I to LC3-II (Figure 3E and Supplementary Figure S3C). Bromo-palmitate, which enters in the cell and blocks mitochondrial β-oxidation, was also without any effect on autophagy by itself (Figure 3E and Supplementary Figure S3C). To definitively eliminate the role of β-oxidation in palmitate-induced autophagy, we incubated INS-1 β cells with a low concentration of palmitate (0.1 mM) in the absence or in the presence of bromo-palmitate. We show that while 0.1 mM palmitate alone induced low conversion of LC3-I to LC3-II (Figure 3F,G; Supplementary Figure S3D), the addition of bromo-palmitate potentiated autophagy induced by low concentrations of palmitate (Figure 3F,G; Supplementary Figure S3D), leading us to conclude that palmitate induces autophagy independently from mitochondrial β-oxidation.

### 3.4. ER Stress Is Not Involved in Palmitate-Induced Autophagy in INS-1 β Cells

**Figure 4:**
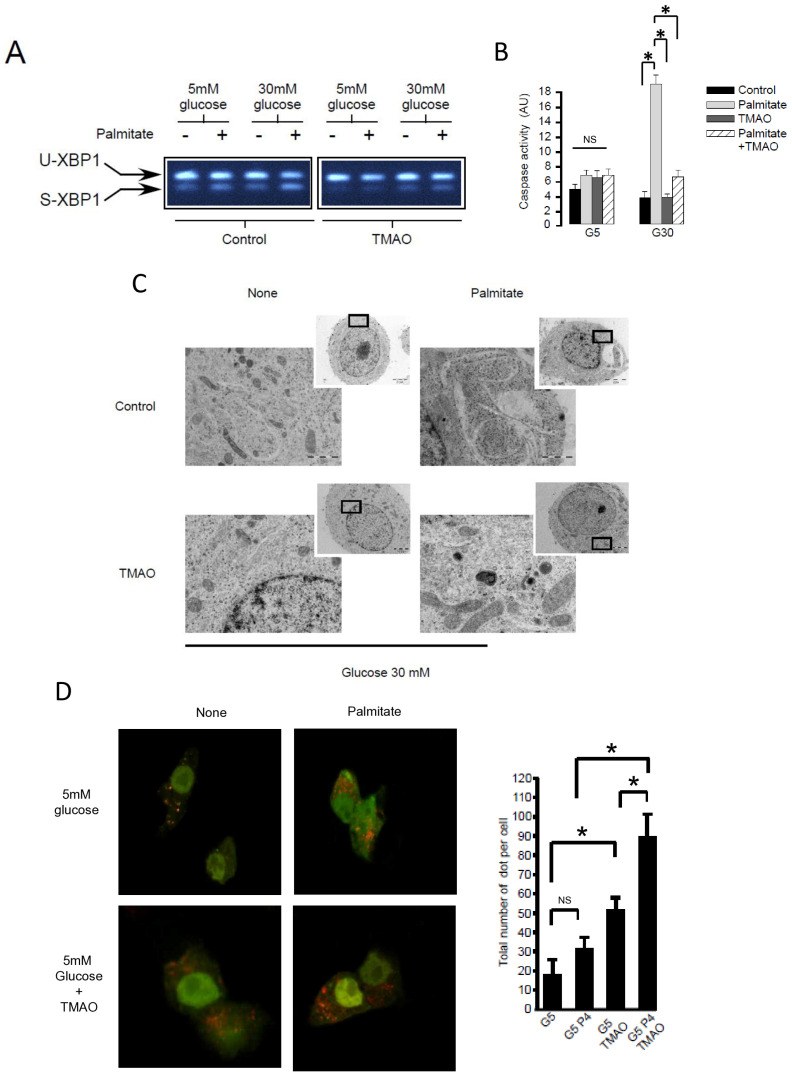

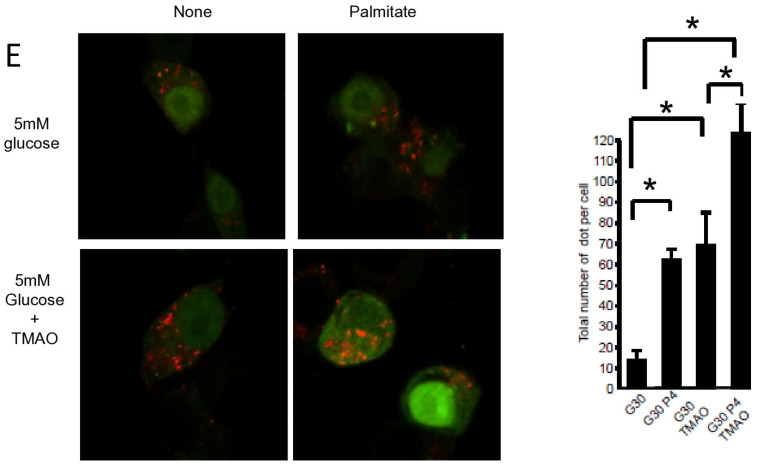
ER stress is not involved in palmitate-induced autophagy in INS-1 β cells. (**A**) Representative image of agarose gel migration of PCR product showing spliced and unspliced XBP-1 (S-XBP1 and U-XBP1, respectively) from INS-1 β cell treated with or without palmitate at 0.4 mM in the presence of glucose at 5 or 30 mM for 6 h. In some conditions, INS-1 cells were treated with 100 mM TMAO. (**B**) Caspase activity expressed as arbitrary units in the same conditions as (**A**) G5: glucose 5 mM and G30: glucose at 30 mM. Results are mean +/− SEM (*n* = 4). * Significant change *p* ≤ 0.01 relative to the control (G5 or G30). (**C**) Electron microscopy photography of INS-1 cells submitted to the different treatments in (**A**). (**D**,**E**) Confocal microscopy analysis of INS-1 β cells transfected with RFP-GFP-LC3 constructed and subsequently treated with the same conditions as (**A**). For each condition, total number of dots (yellow and red) were counted per cell and reported (G5: 5 mM of glucose, G5 TMAO: 5 mM of glucose + TMAO, G5P4 5 mM of glucose, and 0.4 mM of palmitate, G5P4 TMAO: 5 mM of glucose and 0.4 mM of palmitate + TMAO, G30: 30 mM of glucose, G30 TMAO: 30 mM of glucose + TMAO, G30P4: 30 mM of glucose and 0.4 mM of palmitate, G30P4: 30 mM of glucose and 0.4 mM of palmitate + TMAO) for 6 h. Results are the mean +/− SEM (*n* = 4). * Significant change *p* ≤ 0.01 relative to the control (G5 or G30 with or without TMAO).

Pancreatic β cell lipotoxicity has been shown to stimulate ER stress in β cells [25,28]. Nevertheless, the role of ER stress in palmitate-induced β cell autophagy is still controversial [18,21]. Palmitate induced ER stress in INS-1 β cells, reflected by XBP-1 splicing (Figure 4A). TMAO, a chemical chaperone, blocked XBP-1 splicing (Figure 4A) but also blocked luminal swelling associated with ER stress induced by palmitate. (Figure 4C). Moreover, TMAO totally blunted caspase-3/7 activation induced by gluco-lipotoxicity (Figure 4B). Altogether, these data support the primordial role of ER stress in β cell gluco-lipoxicity. Then, we explored the effect of TMAO on autophagic flux induced by palmitate using the mRFP-GFP-LC3 localization in INS-1 β cells (Figure 4D,E). The addition of TMAO with 5 mM glucose increased the total number of yellow and red dots by 2.5-fold (Figure 4D), suggesting that ER stress is a brake to autophagic flux in β cells. In these conditions, palmitate was still able to increase the number of dots in INS-1 β cells (Figure 4D). This rise was due to an increase in red dots (Figure 4D). We performed the same experiment with 30 mM of glucose (Figure 4E), which also led to an increase in the total number of dots (4-fold increase) in the presence of TMAO (Figure 4E). Palmitate with 30 mM glucose still induced an accumulation of red dots in INS-1 β cells (Figure 4E). Therefore, ER stress appears to limit autophagic flux in β cells in basal and gluco-lipotoxic conditions.

### 3.5. Sphingosine Kinase 1 Is Involved in Palmitate-Induced Autophagy in INS-1 β Cells

**Figure 5:**
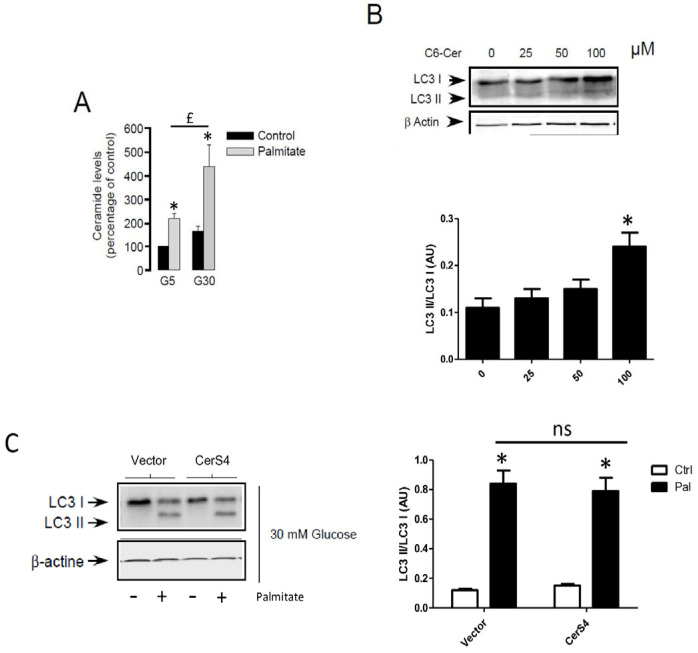
De novo ceramide synthesis is not involved in palmitate-induced autophagy in INS-1 β cells. (**A**) Total ceramide level in the percentage of controls in INS-1 β cells treated with 5 mM of glucose (G5) or 30 mM of glucose (G30) in the presence or absence of palmitate (0.4 mM). Results are the mean +/− SEM (*n* = 3). * Significant change *p* ≤ 0.01 relative to the control (G5 or G30). (**B**) Western blot showing the conversion of LC3-I to LC3-II following treatment of INS-1 β cells by various concentrations of C6-Ceramide for 6 h. Scanning densitometry was performed to quantify changes in LC3-II abundance in cell lysates normalized with LC3-I levels. Results are mean +/− SEM (*n* = 3). * Significant change *p* ≤ 0.01 relative to the control (absence of C6-cer). (**C**) Western blot showing the conversion of LC3-I to LC3-II following 6 h treatment by 0.4 mM palmitate with 30 mM glucose of INS-1 cells transfected with empty or CerS4 plasmid. Scanning densitometry was performed to quantify changes in LC3-II abundance in cell lysates normalized with β-actin levels. Results are the mean +/− SEM (*n* = 3). * Significant change *p* ≤ 0.01 relative to the control; ns: non-significant.

Following its entry into cells, palmitate is activated by an acyl-CoA synthase which is used to produce energy or is transformed into complex lipids such as ceramides. Previous studies have involved the role of sphingolipids in autophagy [26,27]. As previously shown, palmitate induces ceramide accumulation in INS-1 β cells (Figure 5A). In order to determine the role of ceramide in autophagy in β cells, we determined the role of exogenous C6-ceramide on the conversion of LC3-I to LC3-II. Similarly to the MCF-7 breast cancer cells [38], C6-ceramide at a high concentration was able to stimulate autophagy in INS-1 β cells (Figure 5B and Supplementary Figure S4A). We have previously shown that palmitate induces ceramide accumulation through the regulation of ceramide synthase 4 (CerS4) in INS-1 β cells [4]. However, palmitate-induced conversion of LC3-I to LC3-II was not potentiated in INS-1 β cells transiently overexpressing CerS4 (Figure 5C and Supplementary Figure S4B). Altogether, these results suggest that ceramide accumulation is not implied in palmitate-induced autophagic flux in INS-1 β cells.

**Figure 6:**
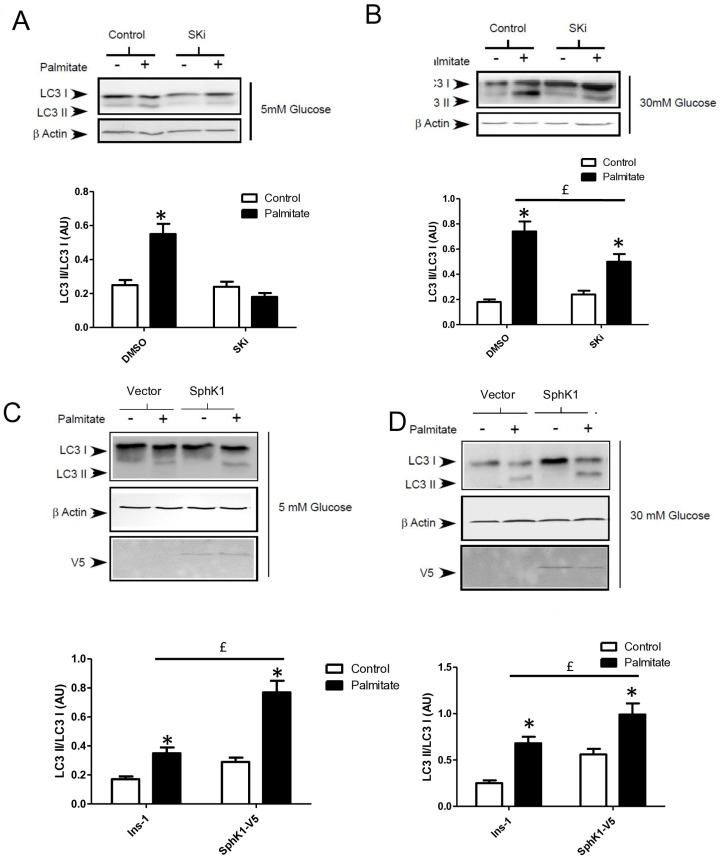

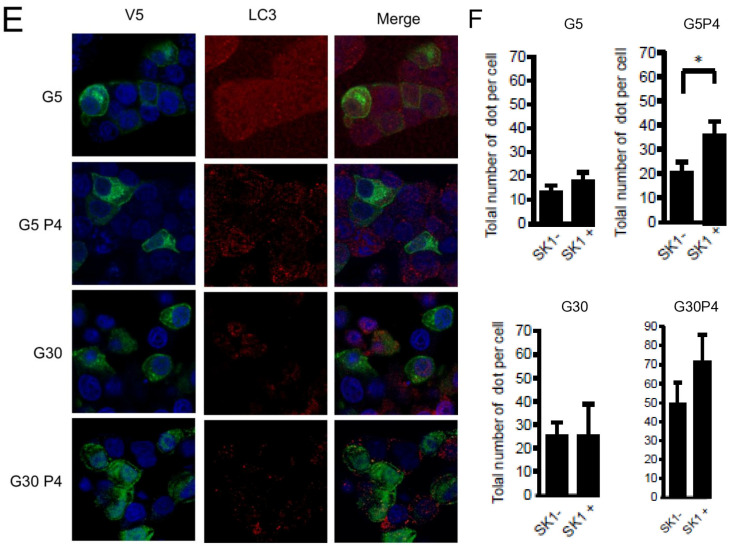
Sphingosine kinase 1 is involved in palmitate-induced autophagy in INS-1 β cells. (**A**,**B**) Representative Western blots showing the conversion of LC3 I to LC3II following treatment by 0.4 mM palmitate in the presence of 5 or 30 mM glucose for 6 h in INS-1 β cells and cells pretreated with 10 μM of SphK1 inhibitor (SKI). Scanning densitometry was performed to quantify changes in LC3-II abundance in cell lysates normalized with LC3-I levels. Results represented as LC3-II/LC3-I (AU) are the mean +/− SEM (*n* = 3). * Significant change *p* ≤ 0.01 relative to the control (absence of palmitate). ^£^ significant change *p* ≤ 0.01 relative to the absence of SKI. (**C**,**D**) Representative Western blots showing the conversion of LC3 I to LC3II following treatment by 0.4 mM palmitate in the presence of 5 or 30 mM glucose for 6 h in vector and V5-SphK1-overexpressing INS-1 cells. Scanning densitometry was performed to quantify changes in LC3-II abundance in cell lysates normalized with β-actin levels. Results represented as LC3-II/LC3-I (AU) are the mean +/− SEM (*n* = 3). * Significant change *p* ≤ 0.01 relative to the control (absence of palmitate). ^£^ significant change *p* ≤ 0.01 relative to the vector-transfected INS-1 cells. (**E**) Confocal microscopy image of INS-1 β cells expressing a V5-tagged SphK1 exposed to 0.4 mM palmitate during 6 h in presence of 5 mM glucose (G5) or 30 mM glucose (G30). Cells were immune stained with anti-LC3 antibody (green) and the nucleus was counter stained with DAPI (blue). (**F**) The number of LC3 dots are recorded in V5-SphK1 negative and positive cells (SK1− and SK1+, respectively). Results are mean +/− SEM (*n* = 4). * Significant change *p* ≤ 0.01 relative for SK1− vs. SK1+.

Gluco-lipotoxicity also stimulated the production of sphingoid base phosphate in INS-1 β cells through the activation of sphingosine kinase 1 activity (SphK1) [28]. In order to elucidate the role of SphK1/S1P in palmitate-induced autophagy in β cells, we first tested an inhibitor of SphK1, SKI [39]. This inhibitor drastically reduced the conversion of LC3I to LC3II in response to palmitate at both 5 and 30 mM glucose in INS-1 β cells (Figure 6A,B; Supplementary Figure S5A,B). Then, we explored LC3-II accumulation in INS-1 β cells stably overexpressing SphK1. We have previously shown that overexpressed SphK1 potentiated the accumulation of sphingoid base phosphate in response to gluco-lipotoxicity [28]. Interestingly, we found that overexpression of SphK1 potentiates conversion of LC3-I to LC3-II in INS-1 β cells treated with palmitate at both 5 and 30 mM glucose (Figure 6C,D; Supplementary Figure S5C,D). To confirm this result, we perform an immunofluorescence detection of LC3-II puncta in INS-1 β cells overexpressing SphK1 submitted to gluco-lipotoxicity. Immunofluorescence with a V5 antibody recognizing SphK1 overexpression showed that INS1 cells taken independently expressed various levels of V5-SphK1 (Figure 6E). We used this diversity in the levels of V5-SphK1 expression in INS-1 cells to explore the role of SphK1 in autophagy induced by palmitate. At low glucose concentrations (5 mM), there was no difference on LC3 dots between low and high SphK1-overexpressing INS-1 cells. Palmitate with 5 mM glucose increased LC3 dots in both low and high SphK1-overexpressing INS-1 cells (Figure 6E). Interestingly, the number of LC3 dots induced by palmitate + 5 mM glucose were increased by two-fold in high SphK1-overexpressing INS-1 cells (Figure 6F). High glucose (30 mM) alone increased LC3 dots in both low and high SphK1-overexpressing INS-1 cells (Figure 6E,F). Again, palmitate + 30 mM glucose increased LC3 dots more efficiently in higher rather than lower SphK1-overexpressing INS-1 cells (Figure 6E,F) without reaching statistical significance.

## 4. Discussion

The globalization of the Westernized diet is suspected to be linked to the increase in T2D [40]. Understanding the role of a high-glucose and high-fat diet in physiology is crucial for our quality of life as well as human health. Our results demonstrate that palmitate with high glucose (gluco-lipotoxic treatment) stimulates autophagy flux in INS-1 pancreatic β cells. The molecular mechanisms involved in the activation of autophagy by excess of palmitate in β cells are still unclear [18,21,41]. Even if palmitate metabolism is crucial to induce autophagy in β cells, here, we provided evidence that de novo ceramide synthesis up-regulated by palmitate [4] is not involved. In contrast, the synthesis of sphingoid base phosphate by SphK1 appears to be a key mediator of β cell autophagy induced by gluco-lipotoxicity.

Gluco-lipotoxicity increases LC3-II levels in INS-1 β cells. This rise, specific to the combined treatment, is also correlated by the increase in autophagosomes observed by electron microscopy. This technique by itself, the gold standard in autophagy research, does not prove the increase in autophagic flux. Treatment with bafilomycin A, a highly potent and specific inhibitor of vacuolar-type H^+^-ATPase, which blocks the fusion of autophagosomes with lysosomes [42], was unable to prevent accumulation of LC3-II in response to gluco-lipotoxicity. The use of the tandem fluorescent protein-tagged LC3 which has both RFP and GFP at the N terminus of LC3 (RFP-GFP-LC3) has also been used to evaluate autophagic flux [21,22,43]. Indeed, RFP-GPF-LC3 protein will emit both red and green fluorescence allowing the visualization of autophagosomes in yellow when images are merged. However, due to the acidic environment of the autolysosome, the GFP fluorescence will be immediately quenched, leaving only the red fluorescent signal unaffected by the low pH [43]. Gluco-lipotoxic treatment demonstrated an increase in both yellow and red dots, proving an increase in autophagic flux in β cells. There is still some discrepancy in the regulation of autophagic flux by gluco-lipotoxicity since both stimulatory and inhibitory effects have been described [18]. This could be related to the time-course of palmitate treatment. We found that palmitate induced accumulation of LC3-II at 6 h but not at 24 h. In agreement with this, previous studies showed that 4–6 h of treatment with palmitate increased autophagic flux, which is decreased over time [18,22]. These results also corroborate the study from Chu et al. (2015), which showed that a high-fat diet increased autophagic flux in pancreatic β cells in vitro and in vivo [24]. Moreover, Bugliani et al. also found an increase in autophagic vesicles in islets from diabetic patients [44]. Taken together, our results suggest that kinetic autophagy plays a pivotal role in response to gluco-lipotoxicity in β cells.

At present, the mechanisms of activation of this early autophagic flux by gluco-lipotoxicity are not known. A review by Lytrivi et al. [8] points out the link between the mitochondrial β oxidation and pancreatic β cell gluco-lipotoxicity. We therefore tested, in our model, if palmitate induces autophagy through the mitochondrial β-oxidation pathway. First, we found that cellular activation of palmitate into palmitoyl-coA by the acetyl-coA synthase is required for inducing autophagy. In agreement with this, methyl-palmitate, which enters in the cell but is not metabolized [45], was unable to induce LC3-II accumulation, supporting the idea that palmitate metabolism is crucial to mediate its autophagic effect. However, inhibition of mitochondrial β oxidation of lipids with etomoxir did not repress the activation of autophagy by gluco-lipotoxicity but rather slightly potentiated it. Similarly, treating β cells with a readily oxidized medium chain fatty acid was unable to increase LC3-II accumulation. These results suggest that gluco-lipotoxicity-induced autophagy requiring palmitate metabolism does not depend on mitochondrial β-oxidation. The slight increasing effect of etomoxir could be due to palmitate metabolism moving toward lipid metabolism.

Previous studies have shown that pancreatic β gluco-lipotoxicity relies on the metabolism of palmitate into lipids such as ceramide [4,9]. Indeed, the de novo synthesis of ceramide promotes the condensation of palmitate with serine in the ER which plays a central role in β cell apoptosis [4]. Moreover, ceramide has been involved in the induction of autophagy in different cell types [46]. Therefore, we hypothesized that the palmitate induced accumulation of ceramide in the ER could be the trigger for the activation of the autophagic flux. In INS-1 β cells, we previously found that CerS4 plays a central role in gluco-lipotoxicity-induced apoptosis [4]. However, overexpression of CerS4 was unable to modulate autophagic flux in β cells. This result contrasted with the study from Gao et al. [47] showing that PEG-ceramide nanomicelles induce an increase in the LC3-II/LC3-I ratio in the murine neuroblastoma metrocyte cell line. This discrepancy seems related to the compartmentalization of ceramide accumulation as multiple studies, including Sakamoto et al., showed the role of ceramide accumulation in the Golgi on its fragmentation independent of other organelles [48]. Similarly, Hou et al. showed that ceramides produced in the mitochondria promote autophagy, and apoptosis in the MFC7 cell line [49].

Pancreatic β cell lipotoxicity has been shown to stimulate ER stress in β cells [25,28]. However, the role of ER stress in palmitate-induced β cell autophagy is still controversial [18,21]. In this study, we found that gluco-lipotoxicity-induced ER stress was evidenced by ER swelling and XBP1 mRNA splicing. Inhibition of ER stress with the chemical chaperone TMAO prevents caspase activation by gluco-lipotoxicty, which strengthens the role of ER stress in β cell death induced by gluco-lipotoxicity [50]. Instead of looking at the effect of ER stress on autophagy initiation (LC3-II accumulation), we looked at its impact on autophagic flux. Surprisingly, inhibition of ER stress by TMAO increased basal autophagic flux in β cells. This effect suggested that ER stress activation by the cell would be a negative control on autophagy. Further experiments will be required to understand the role of the braking effect of ER stress on autophagic flux. We found that inhibition of ER stress is unable to prevent increased autophagic flux induced by gluco-lipotoxicity. Altogether, these results suggest that ER stress could prevent initiation of autophagy but did not regulate the fusion between autophagosomes and lysosomes in response to gluco-lipotoxicity.

Gluco-lipotoxic treatment of INS-1 β cells is associated with de novo ceramide synthesis but also with the production of another sphingolipid, namely the sphingoid base phosphate, which prevents β cell death [28]. Our previous findings showed a predominant role for SphK1 on the onset of apoptosis in INS-1 β cells [28]. In this study, pharmacological inhibition of SphK1 prevents stimulation of autophagy under palmitate treatment. We also found that overexpression of SphK1, which increased sphingoid base phosphate levels in response to the gluco-lipotoxic treatment [28], potentiates the activation of autophagy by palmitate. These results complete the body of literature suggesting the role of S1P and its synthetizing enzyme, SphK1, in autophagy [51,52,53]. The first study showed that SphK1 overexpression increased autophagy in MCF-7 cell lines and was insensitive to FB1 [51]. Interestingly, increased sphingoid base phosphate levels induced by gluco-lipotoxicity are transient in β cells [28] which could also explain the increase in autophagic flux at an early time. It also appeared that palmitate-induced autophagy in the presence of high glucose is less sensitive to the regulation of the SphK1 enzyme. These results suggest that the effect of SphK1 and high glucose on palmitate-mediated autophagic flux could use different pathways. A large body of literature suggests the involvement of autophagy in β cell survival [54]. In these studies, autophagy seems to play a critical role in the regulation of glucose homeostasis controlled by insulin. Likewise, activation of autophagy by inhibition of mTORC1 also increased insulin processing in mouse and human islets [55]. These studies underline the importance of fine regulation of autophagy to promote β cell survival as the deregulation led to increased β cell apoptosis and a reduction in β cell mass [55,56]. The first study linking SphK1 to autophagy showed that overexpression of this enzyme protected cells from cell death [51]. SphK1 and its products are protective against β cell death induced by gluco-lipotoxicity in vitro and in vivo [5]. Our study therefore proposes that SphK1-mediated autophagy is a key mechanism in β cell survival in response to gluco-lipotoxicity. It remains to be determined the signaling pathways involved in the regulation of autophagy by SphK1 in the context of gluco-lipotoxicity. We previously showed that S1P receptors are not involved in the anti-apoptotic effect of SphK1 in INS1 treated by palmitate [28]. Moreover, the selective expression of SphK1 in endoplasmic reticulum had a protective effect against lipotoxicity [28]. Altogether, these data suggest that S1P produced by SphK1 has an intracellular role on the regulation of autophagy induced by palmitate. Recent studies suggested a role of sphingosine in the inhibition of endocytic membrane trafficking and therefore in autophagy. It has also been shown that the recruitment of SphK1 to vesicles reduced sphingosine levels by converting it to S1P which resulted in the stimulation of autophagy [52,57]. Therefore, the rise of SphK1 activity in INS-1 cells induced by palmitate could support a mechanism to favor fusion between autophagosome and lysososme.

Previous studies have connected sphingosine kinase activity to the ability of pancreatic β cells to secrete insulin [58,59]. Moreover, activation or inhibition of autophagy has been shown to regulate insulin homeostasis and secretion [55,60]. Therefore, in the future, it will be interesting to determine the role of the axis SphK1/autophagy on insulin secretion under gluco-lipotoxic conditions.

From a therapeutic standpoint, actual treatment for T2D, excluding diet and exercise, mainly consists of the use of diabetic medications like metformin, a drug discovered nearly 100 years ago and used for T2D treatment since the 1970s. Demonstrating the causality between autophagy, the S1P-SphK1 axis, and β cell gluco-lipotoxicity could open a large avenue of investigation for new drug development that could counteract β cell death. Interestingly, Newton et al. recently described a new SphK1 activator that improved autophagic defects in Niemann–Pick type C1 mutant cells [53].

## Data Availability

Data are available on request to the corresponding author.

## Supplementary Materials

The following supporting information can be downloaded at: https://www.mdpi.com/article/10.3390/cells13070636/s1, Figure S1: Palmitate with high concentration glucose induced autophagy in INS-1β cells; Figure S2: Palmitate with high glucose concentration induced autophagic flux in INS-1β cells; Figure S3: Palmitate induced autophagy requires its metabolism but is independent of its entry in the β-oxidation pathway in INS-1β cells; Figure S4: de novo ceramide synthesis is not involved in palmitate induces autophagy in INS-1β cells; Figure S5: Sphingosine kinase 1 is involved in palmitate induced-autophagy in INS-1β cells.

## Abbreviations

T2D: Type 2 Diabetes, FFA: free fatty acids, ER: endoplasmic reticulum, JNK: c-Jun N-terminal kinases, SphK1: sphingosine kinase 1, si-RNA: small interference RNA, DHS1P: dihydrosphingosine-1-phosphate, S1P: sphingosine-1-phosphate, MEK: Mitogen-activated protein kinase/ERK kinase, EM: electron microscopy, PI3K: Phosphatidylinositol-3-kinase, 3-MA: 3 methyl adenine, BafA1: Bafilomycin A1, LC-CoA: long chain acyl-CoA, DAG: diacylglycerol, TMAO: trimethylamine N-oxide.
