# Supplementary figures and images for: Role of Sphingosine Kinase 1 in Glucolipotoxicity-Induced Early Activation of Autophagy in INS-1 Pancreatic β Cells
Source: Cells. 2024 Apr 5;13(7):636. doi: 10.3390/cells13070636 (PMC11011436; doi:10.3390/cells13070636)

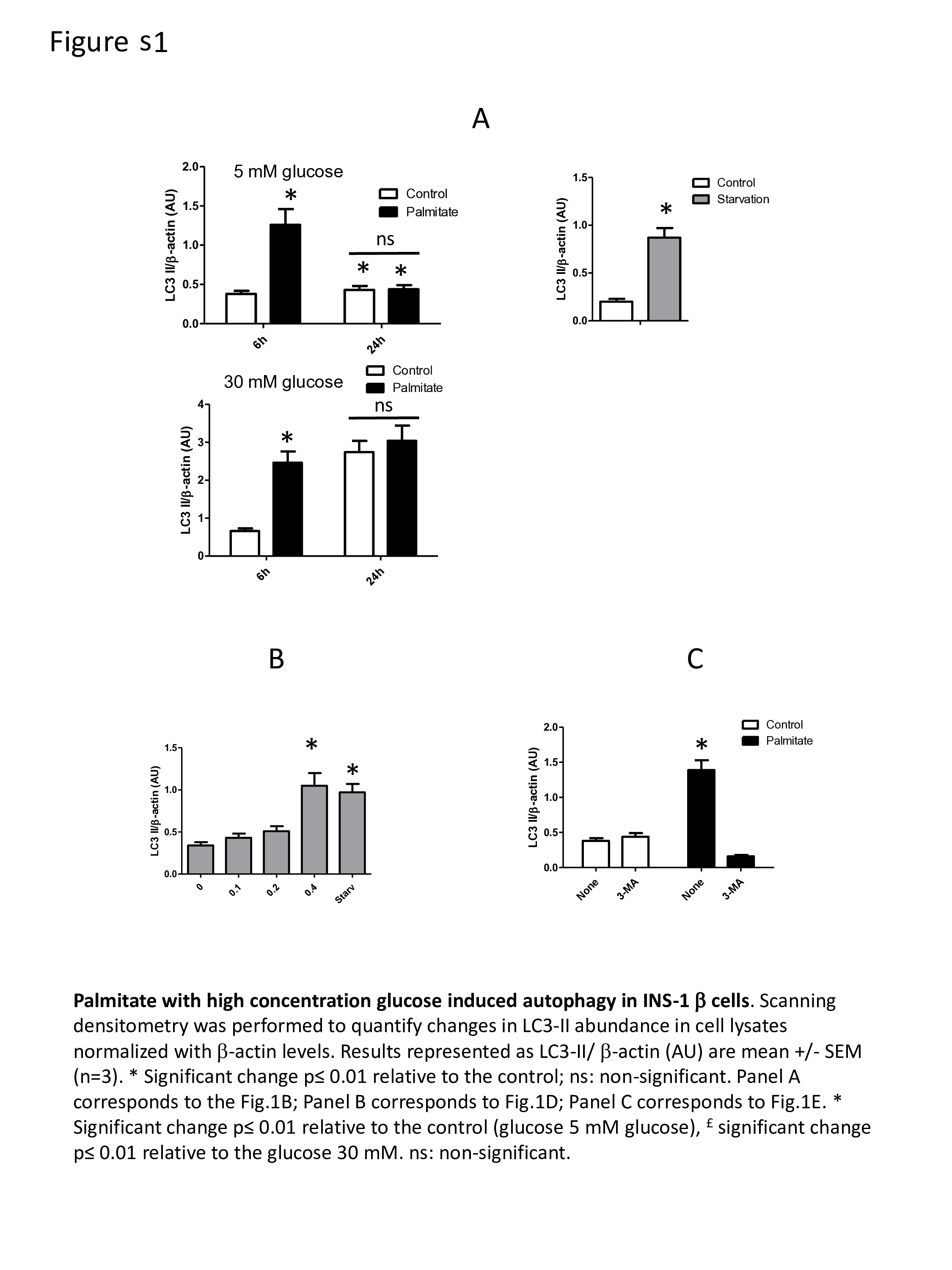

Supplement: Supplementary file 1 [file cells-13-00636-s001.zip › cells-2706966-supplementary-S1.png]

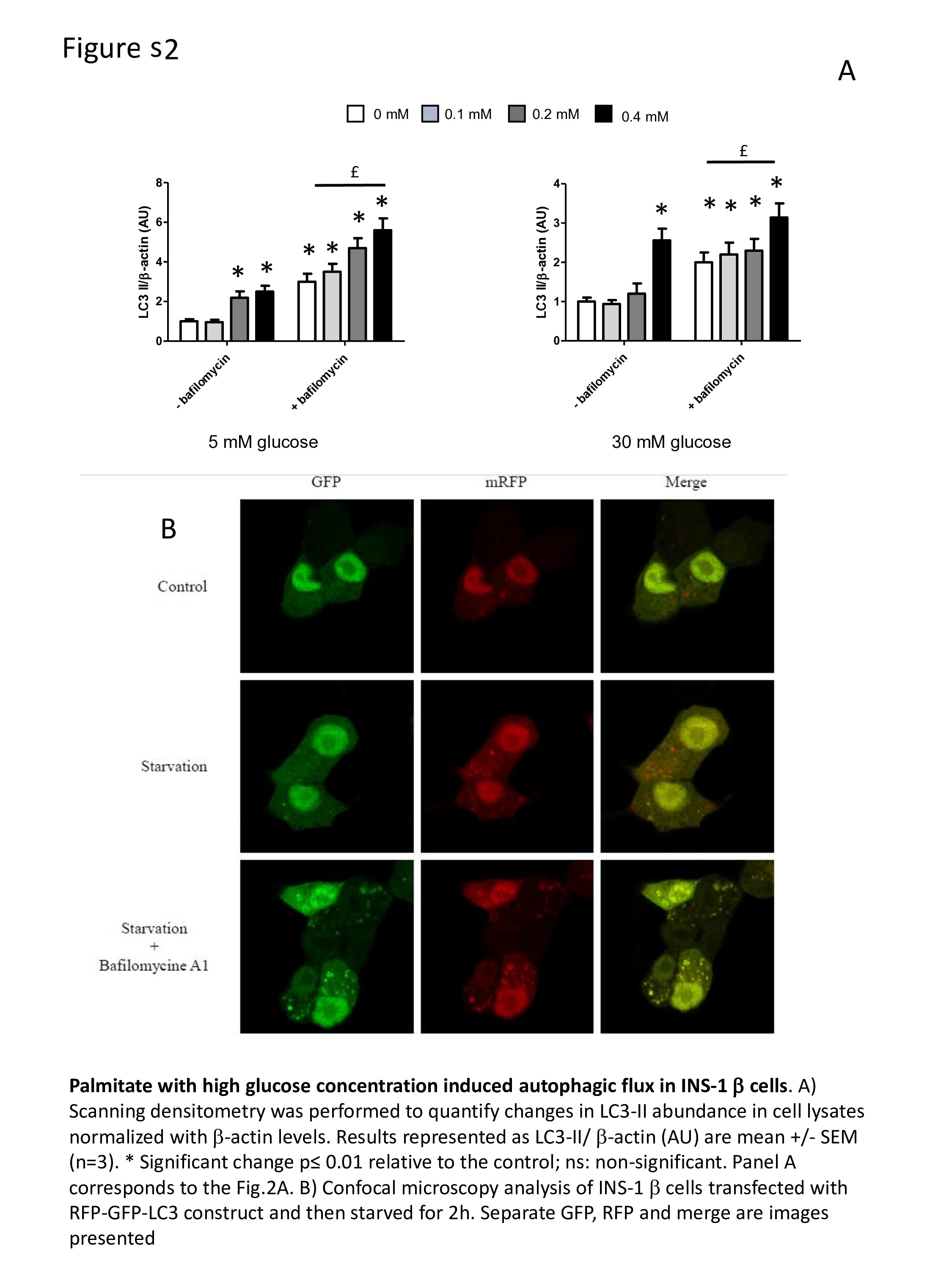

Supplement: Supplementary file 1 [file cells-13-00636-s001.zip › cells-2706966-supplementary-S2.jpg]

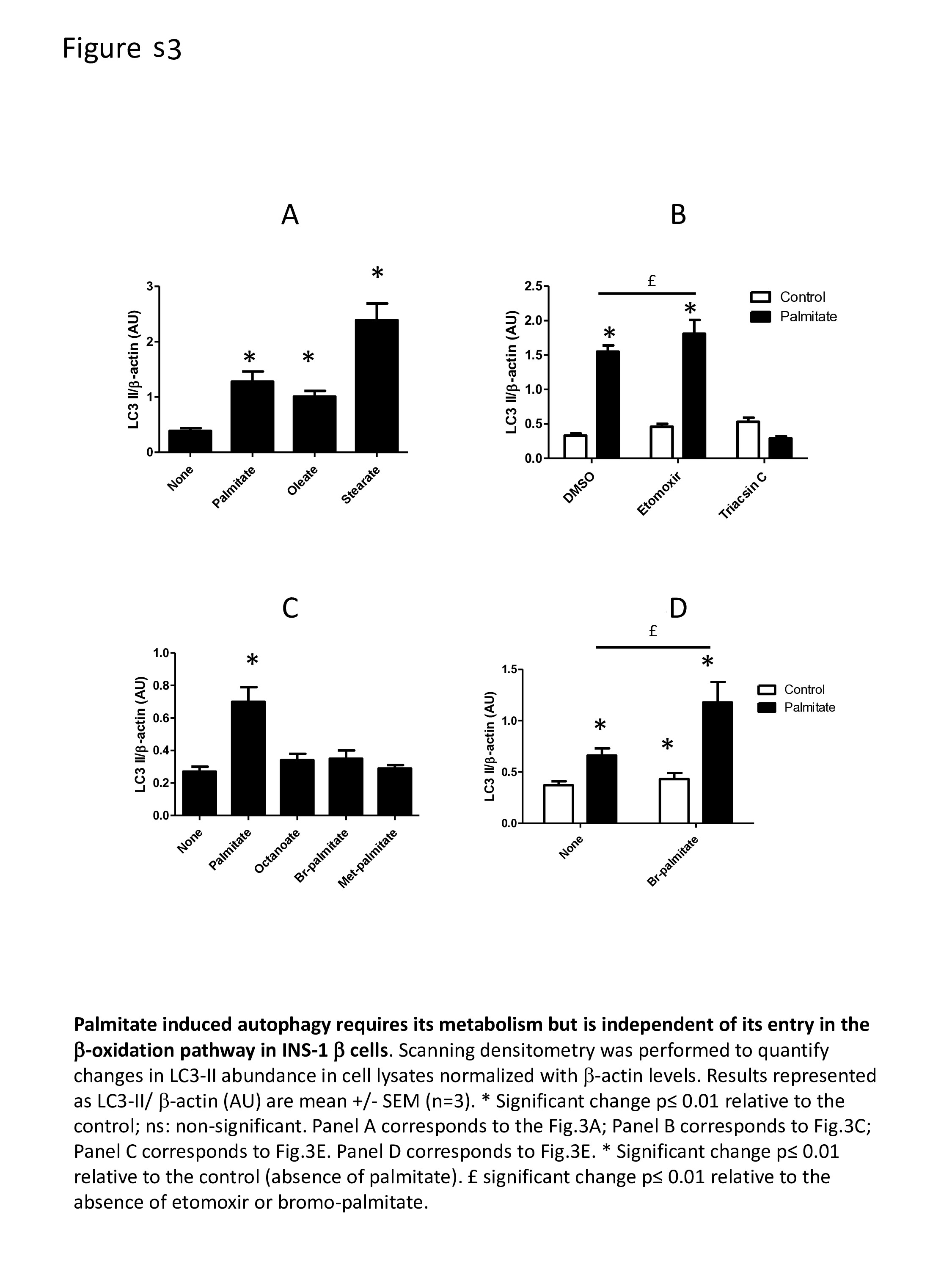

Supplement: Supplementary file 1 [file cells-13-00636-s001.zip › cells-2706966-supplementary-S3.jpg]

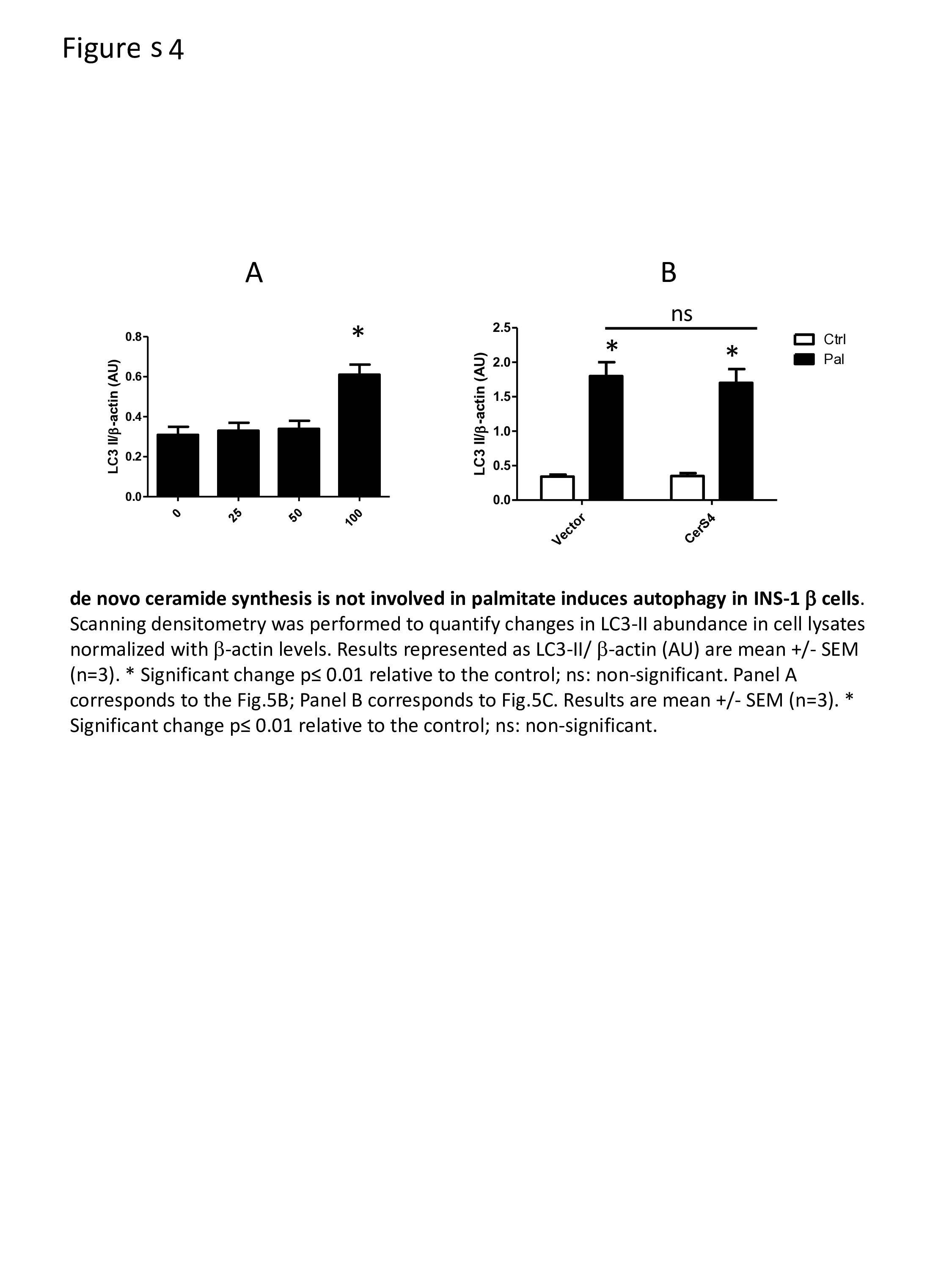

Supplement: Supplementary file 1 [file cells-13-00636-s001.zip › cells-2706966-supplementary-S4.jpg]

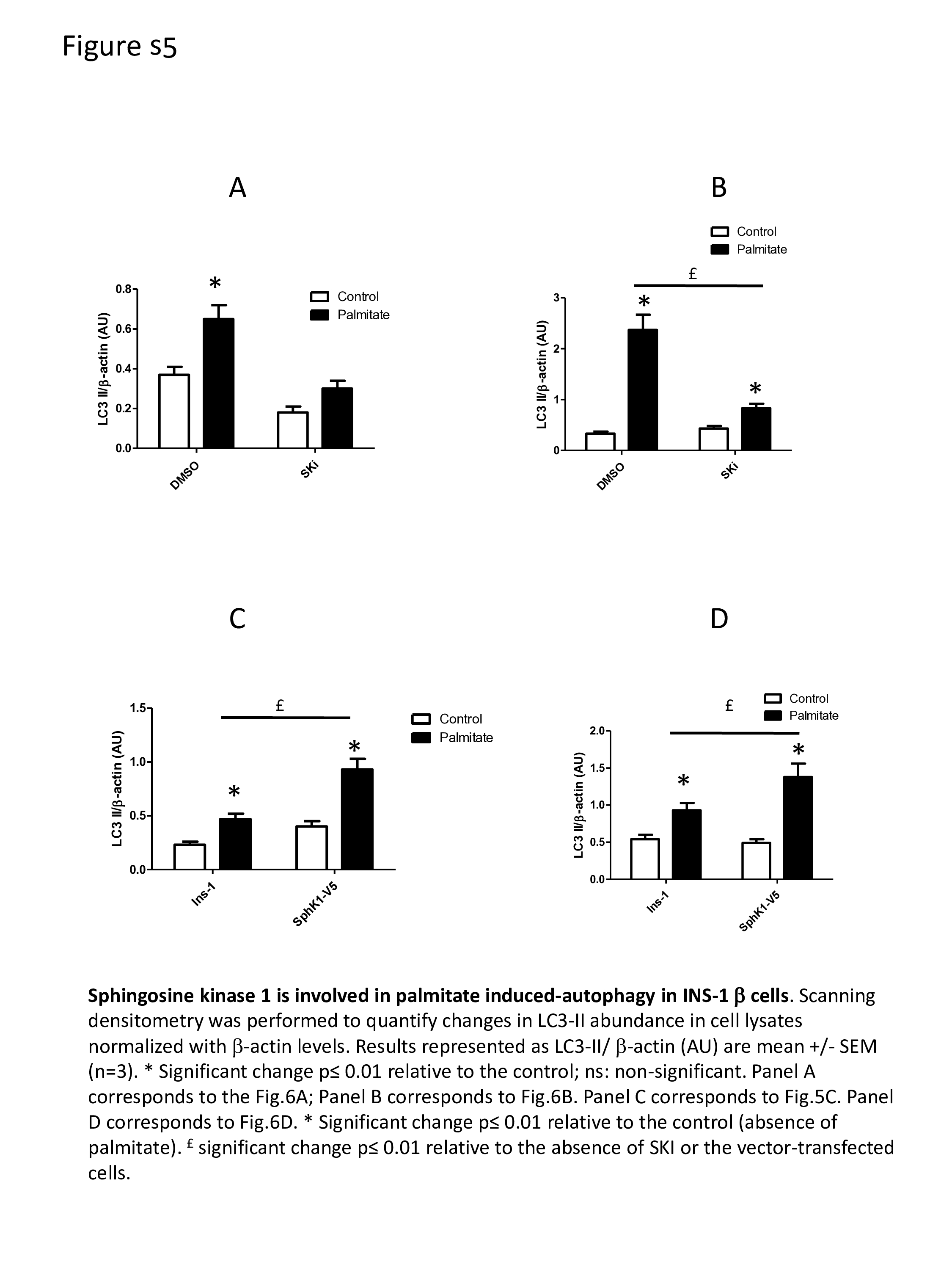

Supplement: Supplementary file 1 [file cells-13-00636-s001.zip › cells-2706966-supplementary-S5.png]
